# Development of Innovative Formulations for Breast Cancer Chemotherapy

**DOI:** 10.3390/cancers12113281

**Published:** 2020-11-06

**Authors:** Ana Isabel Fraguas-Sánchez, Ana Isabel Torres-Suárez

**Affiliations:** Department of Pharmaceutics and Food Technology, Complutense University, 28040 Madrid, Spain; aifraguas@ucm.es

**Keywords:** antibody-drugs conjugates, antineoplastics, HER-2 positive breast cancer, liposomes, microparticles, nanomedicine, polymeric micelles, targeted therapies, triple negative breast cancer

Breast cancer is the most frequent neoplasm in the female population [1]. It is a highly heterogeneous disease mainly categorized into three different subtypes based on the absence or presence of molecular markers for hormone (estrogen and progesterone) and epidermal growth factor (HER-2) receptors. While hormone-receptor positive breast cancer shows, in general, a good prognosis, tumors that do not express any of these receptors, known as triple negative breast cancer, are highly invasive and show a worse prognosis [2,3]. Despite all the advancements undertaken in the last decades for the treatment of breast cancer, especially with the approval of targeted therapies like poly(adenosine diphosphate-ribose) inhibitors (e.g., olaparib and talazoparib) and anti-HER-2 antibodies (e.g., trastuzumab and pertuzumab) that have considerably increased the survival rates of this neoplasm [4,5], breast cancer represents a leading cause of cancer deaths in women worldwide, being a major public health problem.

Among the current strategies for the treatment of breast cancer, chemotherapy represents an adjuvant treatment in most subtypes of this neoplasm and a major treatment option in advanced and triple negative breast tumors [6,7]. Taxanes (paclitaxel and docetaxel), anthracyclines (doxorubicin and epirubicin) are usually administered in the current chemotherapeutic regimens, in combination with a platinum (carboplatin), an antimetabolite (5-Fluorouracil) and/or and alkylating agent (cyclophosphamide). Nevertheless, the high toxicity, low aqueous solubility and rapid in vivo clearance of these chemotherapeutics agents limit their clinical use [8]. For example, taxanes are highly liposoluble drugs that require the use of Cremophor-EL as solubilizing agent, which shows noticeable adverse effects (hypersensitivity, neurotoxicity and nephrotoxicity) that limit the dose [9]. To resolve these challenges, new innovative formulations of chemotherapeutics—most of them based on micro and nanomedicine—are being investigated and developed.

The formulation of chemotherapeutic agents into nanocarriers allows the intravenous administration of these highly liposoluble drugs without using toxic organic solvents, increases their circulation time and favors the selective location of the drug at the tumor mass, decreasing the systemic exposure to the free chemotherapeutic, and, as a consequence, reducing its adverse effects and in some cases even increasing its efficacy.

The selective location of nanoformulations at tumor level can be reached by an active or passive targeting mechanism. The passive targeting of tumors is mainly attributed to the Enhanced Permeability and Retention (EPR) effect [10]. The blood vessels that irrigate tumors show a higher permeability compared with healthy tissues due to the presence of fenestration in the vascular endothelium and the overproduction of vascular mediators (such as bradykinin and vascular endothelial growth factor among others). This higher permeability allows the extravasation, at the tumor area, of the nanoformulations intravenously administered. Moreover, the impaired lymphatic drainage and the slow venous return from the insterstitium favor the permanence of extravasated formulations [11,12,13]. The accumulation of nanoformulations at tumor mass can be improved by an active mechanism, consisting of the incorporation, to the surface of nanocarriers, of ligands specifically recognized by receptors overexpressed on the tumor endothelium or the cancer cells themselves (such as antibodies, antibody fragments, peptides, carbohydrates or vitamins among others) [14,15,16].

Nanomedicines are being widely investigated to improve breast cancer chemotherapy, so that there are already 10 nanomedicines marketed worldwide and 21 nanoformulations under clinical research for the treatment of this type of cancer. Liposomal formulations containing paclitaxel and doxorubicin, are one of the systems most exploited for this purpose. Polymeric micelles and albumin nanoparticles and polymer conjugates have also been developed, showing, in general, a good safety profile. In fact, all approved nanoformulations of paclitaxel show higher maximum tolerated doses than free drug, indicating that its nanoencapsulation is a good strategy to overcome its administration challenges.

Initially, except in the case of Abraxane^®^ -albumin nanoparticles containing paclitaxel- all the nanoformulations that reached clinical stage, were based on passive targeting. However, in the last few years, the development of actively targeted nanotherapies, especially antibody-drug conjugates, has grown considerably, with two marketed formulations and a great number of them under clinical investigation. Most of them are targeted to HER-2 receptors, and, consequently, designed for the treatment of HER-2 positive tumors. However, other targets are also being investigated, such as Trop-2 receptors for the treatment of triple negative breast tumors.

Apart from the nanoformulations, the use of micro-carriers also allows the administration of highly liposoluble antineoplastics without using organic solvents, decreasing the overall toxicity of the formulation compared to the free drug [17,18]. In addition, these devices provide an extended release of the drug after a single administration and, in the case of peptide-based therapy, protection of the active molecule against the action of proteolytic enzymes responsible for its low half-life that conditions its therapeutic utility. In fact, polymeric microparticles containing Leuprolide acetate (Lupron-Depot^®^), parenterally administered every one, three or four months, are already approved for the treatment of several hormone dependent tumors, including breast cancer.

Finally, as we mentioned previously, the chemotherapeutic regimens include the combination of several antineoplastics. The use of nano and micro carriers for their vehiculization also allow the administration of two or more antineoplastics at the same time. This is especially interesting in the case of nanomedicines. The encapsulation of two antineoplastics in the same nanocarrier modifies their biodistribution compared with the free drugs, allowing their location at the tumor site or even inside the cancer cells at the same time, which can increase the antitumor efficacy and prevent the generation of resistances. In this way, several formulations are being investigated, including nanosystems containing paclitaxel and doxorubicin, two major antineoplastics used in breast cancer chemotherapy [19,20,21,22].

In the present Special Issue, researchers are invited to contribute manuscripts reporting original data or updated literature reviews covering the use innovative formulations containing chemotherapeutics for the treatment of any breast cancer subtype.
